# The Agents of Climate Justice in Healthcare

**DOI:** 10.1111/bioe.70106

**Published:** 2026-05-10

**Authors:** Joshua Parker

**Affiliations:** ^1^ Lancaster Medical School Health Innovation One Sir John Fisher Drive Lancaster UK

## Abstract

This paper addresses the critical issue of decarbonising healthcare systems to help combat climate change. I focus on identifying the ‘agents of justice’ responsible for this transformation. Beginning with the claim that healthcare's greenhouse gas emissions cause injustice, the paper assumes that achieving a net zero healthcare system is essential for climate justice. The discussion centres on two prevailing perspectives: one that primarily assigns responsibility to healthcare organisations and another that holds individual healthcare professionals accountable. The paper advocates for a pluralistic approach to responsibility, contending that the complexity and scale of reducing healthcare emissions necessitate allocating responsibilities based on effectiveness. This leads to the identification of two types of responsibility: first‐order responsibilities, which involve direct actions to reduce emissions, and second‐order responsibilities, which involve supporting and ensuring the fulfilment of first‐order duties. The paper clarifies how mitigation responsibilities should be allocated across organisations and individuals by expanding the scope of responsibility to include a broader range of agents, both within and beyond the healthcare sector. By distinguishing between first‐order and second‐order responsibilities, the paper offers a clearer framework for understanding the distribution of obligations in achieving climate justice in healthcare. Ultimately, it underscores that focusing solely on direct mitigation efforts by organisations or clinicians is inadequate, and a more comprehensive, multi‐agent approach is required to effectively decarbonise healthcare systems.

## 1 Introduction

To help avert the threat of climate change, there is a need to decarbonise many sectors and institutions, including healthcare systems. Indeed, numerous healthcare systems globally are committing to reduce their environmental impact^1^ [1]. When reducing healthcare greenhouse gas (GHG) emissions, one important question is who are the duty‐bearers in healthcare's response to climate change? Who are the ‘agents of justice’, responsible for achieving climate justice in healthcare?^2^ [2].

In this paper, I assume that healthcare GHG emissions contribute to injustice and that a net zero healthcare system represents part of healthcare systems achieving climate justice^3^. Healthcare GHG emissions contribute to the threats of climate change [3]. Furthermore, most healthcare GHG emissions come from wealthy countries with technologically advanced healthcare systems, while the greatest effects of climate change are felt by those who contribute the least and are the least equipped to deal with the effects of climate change [4]. At the very least, healthcare must mitigate its GHG emissions to remedy this injustice.

Net zero has become a powerful organising framework for policy responses to climate change across society and many healthcare systems have committed to net zero [5, 6, 7]. Some healthcare systems have also outlined plans on how to become net zero [8]. Hence, we have a better, though not complete, idea of what a decarbonised healthcare system looks like under net zero compared to other potential climate targets. Nevertheless, my arguments regarding the agents of justice should be compatible with alternative targets for achieving climate justice in healthcare, even if I discuss net zero here^4^ [9].

Two camps have emerged in the debate over who has a responsibility to help achieve climate justice in healthcare. They differ on the level at which action on healthcare emissions should be taken. In simple terms, one camp tends to emphasise responsibility at an organisational level [10, 11, 12, 13, 14]. Healthcare organisations like hospitals or the National Health Service (NHS) are the primary duty‐bearer and individual professionals have at most a minor role in healthcare decarbonisation. The reasoning is that organisations have the necessary resources, authority, and systemic influence to implement large‐scale structural changes to healthcare. I say more about the site of action on healthcare emissions and what I mean by organisations later. When I mention organisations in this paper, I am referring to healthcare organisations unless specified otherwise. Others, however, see healthcare professionals as having responsibilities to reduce healthcare emissions [15, 16]. This view argues that professionals should contribute to climate justice through their practice, regardless of organisational efforts. The discussion therefore revolves around an organisational versus individual professional divide.

Consider an example to help highlight the distinction. A polluter pays principle could be invoked to distribute responsibilities for decarbonising healthcare. On this principle, do we say that healthcare organisations are the polluter and thereby liable to pay the costs of addressing emissions? Or does the principle also place responsibilities on doctors, nurses and pharmacists and they too should make decisions in line with a polluter pays principle?

Many healthcare systems are already committed to decarbonise and are formulating policies in light of this commitment. Shifting healthcare systems away from the emissions that they have relied on to deliver the benefits of healthcare represents an enormous challenge and is thought to require a radical transformation of healthcare [17]. How healthcare reconciles meeting its goals with the burdens of reinvention to low‐carbon systems raises questions of distributive justice [18]. One question, is where principles of justice that are intended to govern healthcare decarbonisation apply? What is the site of climate justice in healthcare? Whilst the organisational vs. individual divide relies on this question of the site of justice, to date this framing is absent from the debate and so its implications are unexplored. The simplification of the organisational vs. individual divide overshadows the scale and complexity of decarbonising healthcare. Even if we accept that both organisational and individual‐level action is necessary to sufficiently decarbonise healthcare, it is still an open question how to fairly allocate responsibilities for realising climate justice in healthcare across various actors. Furthermore, a unitary account of organisations as the locus of mitigation responsibilities for healthcare is lacking in the debate and these metaphysical issues have important moral consequences. This paper contributes to the debate by clarifying the site of justice and how it relates to who the agents of climate justice in healthcare are given the challenge facing healthcare in the transition to low‐carbon environmentally sustainable systems. Understanding this issue is critical for making progress on how healthcare systems contribute to addressing climate change.

This paper defends a plural account of responsibilities to reduce healthcare emissions. I argue that the complexity and scale of reducing healthcare emissions means that responsibilities should be allocated based on agents' capacity to reduce emissions. A focus on effectiveness leads one to identify two distinct responsibilities: first‐order and second‐order responsibilities. First‐order responsibilities are necessary to directly reduce healthcare emissions, and I argue that numerous agents, not just healthcare organisations nor clinicians, are necessary to successfully achieve a net zero healthcare system. In other words, my view is that whilst organisational and clinician action is necessary to achieve climate justice in healthcare, it is insufficient. To be effective others, including but not limited to healthcare organisations and professionals, must also take action to ensure that first‐order responsibilities are fulfilled. These are second‐order responsibilities. Hence my arguments further the debate by broadening the scope of the tasks required to decarbonise healthcare from first‐order only to also include second‐order responsibilities, thereby moving beyond an organisation vs. individual dichotomy and considering how responsibility is shared amongst various agents. Ultimately, I underscore that focusing solely on direct mitigation efforts by organisations or clinicians is inadequate, and a more comprehensive, multi‐agent approach is required to effectively decarbonise healthcare systems.

The paper is structured as follows. To begin I describe how healthcare systems can reduce emissions. Net zero healthcare requires transformative change across healthcare and various tasks are necessary to achieve this. This context foregrounds the analysis of the site of justice and the agents of justice. Next, I discuss the organisational vs. individual divide in terms of the site of justice. I clarify what I mean by healthcare organisations and argue that although the site of justice is healthcare organisations, this need not limit us to excluding action from individuals like professionals (or patients). I then make the case that responsibilities to reduce healthcare emissions should be allocated based on agents' power to effect change. Numerous actions across the health sector and beyond are required to reach a net zero healthcare system necessitating action beyond the organisation‐clinician divide. I rely on a distinction between first‐ and second‐order responsibilities to highlight the variety of methods necessary to reduce healthcare emissions. Finally, I discuss how various agents including governments, non‐state actors, healthcare organisations, clinicians and patients have first‐ and second‐order responsibilities to achieve climate justice in healthcare.

## 2 Net Zero Healthcare Systems

Disentangling healthcare emissions is complex. Modern healthcare systems have a complex structure and organisational pattern, and the provision of healthcare is complicated by this as well as by the multifaceted nature of the problems that healthcare systems face. To establish the carbon footprint of healthcare scientists must identify the boundaries of healthcare and then determine all the structures and processes required to provide healthcare. In other words, we need to say what counts as healthcare as well as drilling down to understand how healthcare works to track healthcare emissions. With this in place, life‐cycle assessments of healthcare products and processes can begin to reveal the carbon footprint of healthcare [19].

Global appraisals of healthcare's carbon footprint range between 4% and 6% of all emissions in 2016^5^ [4, 20, 21]. Healthcare emissions also vary by country with the United States (27%), China (17%) and the European Union (12%) accounting for more than half of the world's healthcare carbon footprint [17, p. 34]. In order to get an idea of where healthcare emissions come from, I focus on the NHS as there is reasonable data. The proportion of emissions from various sources in the NHS is broadly similar to global estimates however [17, p. 34].

To gauge the emissions of the NHS in England, Tennison and colleagues use a hybrid method, combining top‐down and bottom‐up approaches [22].This helps to provide the most accurate and comprehensive measures to date. In accordance with the Greenhouse Gas Protocol framework, they divide their findings across three scopes: scope one refers to emissions under direct control of healthcare (17% of emissions) like anaesthetic gases, scope two emissions are essentially purchased electricity (12% of emissions) and scope three contains all other indirect emissions (71% of emissions) for example those embedded in medical supply chains. In 2019, the total carbon footprint of the NHS in England was 25 megatons of CO_2_ equivalent (CO_2_e). Predictably, most emissions come from the supply chain (62% [15.6 megatons]) followed by the delivery of care (24% [6.1 megatons]). With scope three accounting for the lion's share of healthcare emissions, it is apparent that action beyond the NHS will be necessary to meet the NHS in England's target of an 80% reduction in emissions by 2039 [8].

Decarbonising healthcare entails changing how, where and what healthcare is provided [23]. As all care contributes, at least indirectly, to emissions most healthcare emissions are embedded in the complicated pathways and processes that make up the delivery of modern healthcare [24]. Consequently, it is uncommon to find interventions with a substantial carbon footprint that can be easily identified and substituted. Exceptions include metered‐dose inhalers and volatile anaesthetic gases, which rely on greenhouse gases and so can be switched for alternatives [15, 18]. Overall, achieving net zero healthcare involves reducing demand for high‐carbon care, minimising emissions from care delivery, decarbonising the healthcare supply chain and offsetting any residual emissions [19].

A systematic blueprint for a net zero healthcare system is lacking [25]. However, the NHS has laid out the most detailed and comprehensive plan to achieve net zero to date [8]. Transforming healthcare systems to net zero is thought to be highly distributive involving system‐wide transformative change. Key areas of decarbonisation include: the supply chain, energy use, transportation, food systems, waste management, transformative low‐carbon models of care, as well as changing the culture, norms and financial incentives that structure and organise healthcare [19, 25]. Healthcare without Harm, for instance list: power healthcare with 100% clean renewable energy; invest in zero emissions buildings and infrastructure; transition to zero emissions, sustainable travel, and transport; provide healthy, sustainably grown food and support climate‐resilient agriculture; incentivise and produce low‐carbon pharmaceuticals; implement circular healthcare and sustainable healthcare waste management; and, establish greater health system efficiency [17, p. 4].

I do not suggest that these are the definitive list. Rather, I hope to give a sense of the scale of reducing healthcare emissions. Numerous tasks are therefore necessary to achieve sufficient overall carbon reductions in healthcare (Table 1) [8, 18, 25, 26]. With an outline of the complicated tasks necessary to put us on a path to a net zero healthcare system, the question is who has what responsibilities to achieve such tasks?

**Table 1:** Key target areas for achieving a net zero healthcare system as well as the important tasks to mitigate emissions.

Target area for emissions reduction	Tasks to achieve emissions reduction
Supply chain	Green supply chain sourcing of low greenhouse gas products, e.g., pharmaceuticals with lower manufacturing energy requirements
	Local procurement where possible
	Avoid plastic products
	Reduce downstream demand
	Use of purchasing power and relationships with suppliers to set emissions reduction targets and environmental standards
Infrastructure: Buildings, facilities, and energy	Convert gas boilers to heat pumps
	Electrify buildings
	On‐site wind power and photovoltaics
	Retrofit existing buildings to provide insulation and increase energy efficiency
	Water collection and water recycling facilities
	Enhance lighting efficiency
	Green building design
	Shutdown checklists in energy‐intensive areas like theatres
Transportation	Reduce transport requirements: reduce staff and patient travel through active travel, carpooling etc.
	Carbon‐efficient fleet vehicles
	Community‐based services
Food systems	Reduce food waste
	Low‐carbon foods and packaging
Waste management	Reduce reliance on single‐use consumables
	Water efficiency projects and reduce wastewater and solid waste
Low‐carbon models of care	Disease prevention and public health promotion through tackling the social determinants of health, patient education, and public health campaigns.
	Improving management of chronic disease
	Prevent unnecessary and low‐value healthcare through for example reducing overtreatment and overdiagnosis. Reduced wasteful and inefficient care practices.
	Low‐carbon alternatives and green prescribing, for example metered‐dose inhalers and volatile anaesthetic gases. Social prescribing.
	Innovative technologies like telemedicine and remote healthcare. Digital health technologies like eHealth and mHealth apps. Capture technologies for volatile anaesthetics.
	Move care out of acute hospitals and provide more healthcare in the community.

*Note:* Adapted from: NHS England (2020), Tennison et al. (2021), Salas et al. (2020), Issa et al. (2024), Schroeder et al. (2012).

## 3 The Site of Climate Justice in Healthcare

The introduction mentioned that one aspect of the debate surrounding healthcare decarbonisation is where the relevant responsibilities fall. This is closely related to, and shares features with, another issue in political philosophy regarding the site of justice. The site of justice is concerned with the kinds of entities or practices to which fundamental principles of justice apply; whether to institutions, or to individual's actions and choices, or something else [27].

One key debate regarding the site of justice is whether this includes personal conduct or not [27, 28, 29, 30]. John Rawls, for example, claimed that the site of justice was the major social institutions in a society [31]. When principles of justice are applied to healthcare to allocate mitigation responsibilities, the question arises as to the level at which the principles apply. Do principles of justice apply at the broad level of healthcare as an organisation changing the character of healthcare as a whole, or do they apply to the distribution of healthcare activities or services, or even to individual conduct and professional practice altering the choices that doctors and patients make, or is it all of the above? The site of justice shapes the scope of mitigation responsibilities and hence dictates who the agents of justice are [32].

### 3.1 Division of Labour and the Site of Justice

In *A Theory of Justice*, Rawls limited the site of justice only to the basic structure and excluded people's day‐to‐day actions and decisions: ‘the principles of justice for institutions must not be confused with principles which apply to individuals and their actions in particular circumstances. These two kinds of principles apply to different subjects and must be discussed separately.’ [31, p. 47] For Rawls, there is a division of labour then between the moral demands on institutions and the moral demands in personal life [30]. The reason that Rawls focuses on the basic structure is because it has a profound influence over people's life chances, sets the conditions for social cooperation and has powers of coercion. However, Rawls goes on to clarify that whilst individuals have so‐called ‘natural duties’ to establish and support just institutions [31, p. 99], as well as duties in their interactions with institutions or roles they might occupy within an institution, beyond this they should be left free to pursue their own ends unencumbered from the demands of egalitarian justice [33].

The idea of a division of labour is reflected in arguments forwarded by those who think that it should primarily fall to healthcare institutions to decarbonise healthcare. This division is made by several scholars. Yoann Della Croce and Ophelia Nicole‐Berva argue that, ‘these two levels [the “meso level” of institutions and ‘micro level’ of healthcare professionals] are interdependent at multiple scales regarding practical implementations, [but] they require *different theoretical foundations and separate analyses* of how their duties toward climate protection are justified. [my emphasis]’ [14] That is, different principles of climate justice apply to institutions and individuals. Others, similarly, worry about overemphasising the individual level and overlooking systemic change. Gabby Samuel and colleagues write, ‘Focusing here on the doctor–patient dialogue as the locus of change draws attention away from structural mechanisms such as regulatory approaches, which, in a functioning regulatory system, would have the potential to bring about quicker and longer‐lasting reform.’ [11] A parallel concern is raised by Sapfo Lignou and James Hart who argue that ‘policies should be developed to dictate when and how climate considerations limit available individual patient care enabling clinicians to focus on assessing the clinical benefits of particular interventions and ensuring compliance with these policies.’ [12] The idea is that the demands of climate justice operate primarily at an institutional‐level and do not fall to individuals. Healthcare organisations should be designed such that they realise climate justice through policies which leave healthcare professionals and patients unencumbered by environmental issues and able to focus on individual patient care.

Although these scholars discuss the institutional level, I distinguish institutions from organisations similarly to Douglass North, who describes institutions as ‘the rules of the game’ and organisations as ‘the players’ who operate within the framework of institutions [34]. I take it that these authors are referring to healthcare organisations since it is less clear who the target of mitigation responsibilities are when they speak of institutions, unlike organisations. Organisations are understood as a ‘collective agent that involves a large number of people who realise a structure that coordinates divided labour via rules and hierarchical command relations, guided by a collective decision‐making procedure.’ [35] Organisations exist in the world, like banks and universities, unlike institutions. Institutions, on the other hand, are the formal and informal rules and norms that organise social, political and economic relations [34]. Institutions capture broader and more abstract phenomena like marriage and the market which are not organisations, even if they bear on them. Sometimes, organizations can be grouped into meta‐organizations, so a meta‐organization is basically just a type of organization, one formed from other types of organization. Meta‐organisations may well be relevant for decarbonising healthcare since other non‐healthcare organisations could have responsibilities to assist in decarbonising healthcare. Hospitals, GP surgeries and the ambulance service are all organisations, but they can also be part of a meta‐organisation like the NHS.

Organisations, unlike institutions, are formed from identifiable people who occupy roles and stand in certain relations. The result is that there is a boundary between members of an organisation, and non‐members. Two main factors contribute to separating healthcare organisations into unitary entities with a distinct membership: their structure and purpose. Organisations are composed of people and physical elements who together form a structure. A structure is an abstract entity that describes the relations between the roles that people occupy and physical goods like buildings, medical instruments, ambulances and the like [36, 37]. People occupy various roles in healthcare organisations: doctors, nurses, physiotherapists, pharmacists, radiographers, porters, managers, accountants, administrators and so forth. Within these roles, people interact with one another, with patients and with physical goods like CT scanners, buildings, medicines, and others in complex relations that form structures. It is from these structures that healthcare resources and goods are distributed through activities and services. The roles, material elements and relations will define different healthcare organisation types. Healthcare's purpose tends to surround protecting and promoting health, as well as preventing suffering, care of the dying and so forth.

This paper assumes that when these authors discuss climate action at the organisational level, they view an organisation as a collective agent for the purpose of allocting mitigation responsibilities. Collective agents are composed of individuals who form a unit for the purposes of making decisions and taking action, and are guided by a decision‐making procedure. Collective agents can have goals, make decisions, and act in the world. There is an important question about the relationship between organisations and their members such that an organisation is considered as an agent. This is important for assigning responsibilities. If we say that the NHS is responsible for providing COVID vaccinations, there is a sense in which we assign responsibility for this particular task to the NHS as a collective agent above and beyond the components that make up the NHS. A further account is needed of the internal processes and relationships that make sense of the idea of holding the NHS responsible above and beyond holding individual members responsible. Furthermore, this account needs to explain how the NHS takes action in the world, say to provide COVID vaccines, through its members without simply being reducible to the aggregated actions of individuals. Philosophers answer these by defending various accounts of collective agency [38]. This paper does not defend a particular account of collective agency, rather it assumes that since the NHS is highly organised and unified, with goals and decision‐making procedures it can be considered as a collective agent for the purpose of assigning responsibilities.

Given the complexities of decarbonising healthcare apparent from the descriptions above (Table 1 especially), many argue it is overwhelmingly preferable for organisations to take a central role in decarbonising healthcare rather than leaving it to the uncoordinated efforts of motivated clinicians and climate conscious patients. This is the division of labour mentioned above. The issue is whether the business of securing climate justice in healthcare should primarily occur away from clinicians and patients. Clinicians, we might think, are better left to what they do best: making diagnosis, treating patients, preventing illness and so forth. Patients too, should be left free to make healthcare decisions by their own lights unburdened from the worries of a net zero healthcare system. In other words, one reason to divide responsibilities in this way is to prevent them being too burdensome on individuals and because organisations are more effective.

I agree with the proponents of the organisational view that structural change is necessary to decarbonise healthcare. Since structures are necessary to allocate healthcare goods and services, one goal of mitigation in healthcare is to alter those structures such that healthcare goods and services and provided in ways that minimise emissions. What is unclear from the proponents of organisational and structural change is how to allocate responsibilities to change organisational structures. In particular, there is a question of the metaphysical relations between organisations and their members that allows organisationalists to distinguish between climatic responsibilities for some and not for others, namely clinicians. Since this relationship is untheorized, when organisations are the target of mitigation responsibilities, it can seem that organisations are somehow separate to the individuals that form the structures from which organisations are composed in the division of mitigation responsibilities. As I have sketched organisations, the structures that form organisations involve relationships between people including clinicians as they occupy roles, so organisations are not separate to individuals. Rather, through structures, organisations are given unity as collective agents whilst recognising they are formed from many individuals. The question for proponents of the organisational view is how healthcare structures, of which clinicians are a part of, can be altered whilst exempting the clinician‐patient relationship? In what follows I also take an organisational stance, but argue that principles of climate justice in healthcare apply at multiple levels and to several actors to change healthcare structures by allocating the tasks listed above to help transform healthcare to become net zero.

### 3.2 Ability to Pay

Typically, three principles are used to help allocate mitigation responsibilities: a polluter pays principle, beneficiary pays principle or an ability to pay principle [39, 40]. Polluter pays allocates mitigation burdens on the basis of contribution to the problem; those who play a causal role in emissions have a remedial responsibility to tackle them. A beneficiary pays principle allocates responsibilities to those who benefit from the injustice. Ability to pay is interested in who has the greatest capacity to remedy an injustice. Here I rely on ability to pay.

Let us first discuss the polluter pays principle because this principle has much intuitive appeal in allocating responsibility^6^. Despite its appeal, the polluter pays principle is inadequate for determining the agents of achieving climate justice in healthcare. Considering the scale of decarbonising healthcare, a contribution‐based way of allocating responsibility may struggle in the face of the causal complexity. Emissions that are embedded in complex healthcare systems and processes can make it tricky to single out *the* polluter. Consider the emissions from an inpatient hospital stay. Per day in a hospital bed, there are estimated to be 125 kg of CO_2_e [22]. This derives from: buildings and energy, water and waste, staff travel, medical and non‐medical equipment, pharmaceuticals and chemicals, and other procurement like food. So, who is the polluter when it comes to that 125 kg of CO_2_e per bed‐day? It seems to me that it is impossible, or at least extremely challenging, to isolate the polluter. But without an identified polluter, it is hard to say who pays.

A second concern with the polluter pays principle is that reducing the emissions of healthcare requires action from agents outside of healthcare. It is thought, for example, that to achieve net zero healthcare demand must reduce, especially for highly specialist and technologically advanced interventions. One important way to mitigate healthcare emissions is to look upstream and tackle the social determinants of health. Both individual practitioners and healthcare organisations are extremely limited in what they can do to directly affect the social determinants of health, however. Rather, governments and other state and non‐state actors are needed to tackle poverty, provide adequate education, improve housing and working conditions, and so forth. Addressing the social determinants of health is clearly important and not just because of its impact on healthcare emissions. But, in looking to allocate responsibility for mitigating healthcare emissions, it's difficult to see how a principle based on contribution to a problem, like a polluter pays principle, takes us to a responsibility for governments and others to address the social determinants of health. If polluters pay, it is not clear how a government, for example, is a polluter in respect of failing to tackle the social determinants of health, meaning there is a greater demand for healthcare, which in turn generates healthcare emissions. An account for identifying the agents of climate justice in healthcare needs to attend to the full scope of actions necessary to mitigate healthcare emissions as well as all the potential agents who can perform these.

A different approach to a contribution‐based principle like polluter pays is to look at the various and complex tasks involved in mitigating healthcare emissions and ask who can most effectively achieve those tasks? The urgency of averting dangerous climate change and healthcare's important position in reducing emissions offers one reason to allocate responsibility based on effectiveness. Since we have a broad picture of what needs to be done to achieve net zero healthcare, it makes sense to focus on effectiveness when dividing up responsibilities. Furthermore, as I detail below, attending to the project decarbonising healthcare from the perspective of effectiveness widens the scope of the potential agents of justice from just individuals versus healthcare organisations.

Ability to pay is a forward‐looking principle that assigns responsibilities to address a problem based on agents' capacity [41]. In terms of climate change, capacity is usually understood as being wealthy. But for healthcare systems, we can understand ability to pay as reflecting what agents can do to decarbonise, which is in turn shaped by their effectiveness and the costs to them in contributing to solving this problem. I do not offer a thorough defence of ability to pay as the most appropriate principle for reconciling how healthcare achieves its goals with decarbonising such that healthcare does its fair share. I undertake this task elsewhere [18, 42]. For my purposes, the key advantage of ability to pay is that it is sensitive to the burdens of mitigation for agents. Since I take it that the main site of justice is organisational, then ability to pay regulates how healthcare systems should decarbonise and this extends to what healthcare professionals, patients and wider society should do in their interactions with healthcare and in their duties to establish and uphold climate justice in healthcare^7^.

As Table 1 shows, reducing healthcare emissions is a complex task that involves numerous changes to what care is provided, where it is delivered, and how it is administered. I argue that both healthcare organisations and clinicians play essential roles in effectively achieving the transformative changes needed for a net zero healthcare system. However, their efforts alone are not enough. Since, on my view, responsibilities for achieving climate justice in healthcare should be allocated based on effectiveness, responsibilities should not only focus on directly reducing emissions but also include actions that support emissions reduction. In particular, the agents of justice should be recognised by their capacities to effectively decarbonise healthcare and so we need to look at a whole range of actions that can be taken. Furthermore, the range of agents responsible should be broadened to include more than just healthcare organisations or clinicians. To better understand who the agents of climate justice in healthcare are, and how responsibilities are allocated I distinguish between first‐order and second‐order responsibilities.

## 4 The Agents of Climate Justice in Healthcare

Distinguishing between first‐order and second‐order responsibilities is crucial for effectively allocating mitigation duties [43]. First‐order responsibilities involve the direct obligations agents have to reduce emissions, based on their specific capabilities. These responsibilities stem from the unique powers and roles that agents hold. For example, those in leadership positions, with decision‐making authority, or relevant expertise, are naturally suited to taking direct action at the level of organisations, such as installing solar panels, switching energy suppliers, or upgrading the ambulance fleet to low‐emission vehicles. However, an agent's capacity to act is also shaped by the realities they face. They may, for example, have other important commitments or responsibilities which limit their ability to fully engage in emissions reduction.

On the other hand, second‐order responsibilities focus on supporting and ensuring the success of first‐order actions. Achieving a net‐zero healthcare system depends not just on those directly reducing emissions, but also on those who facilitate, enforce, and promote these efforts. Simon Caney identifies a number of examples of second‐order responsibilities, including: enforcing compliance, creating incentives, establishing norms, reducing resistance to effective climate policies, and civil disobedience [43, pp. 136–139].

As will become clear in the discussion below, first‐order and second‐order responsibilities cut across and extend beyond the organisational versus individual divide previously discussed. That is, different kinds of actor can take on first‐ and second‐order responsibilities depending on who would be most effective, and sometimes the same actor can have both kinds of responsibilities in different circumstances. Although I claim that an ability to pay principle should govern how healthcare systems decarbonise, this leaves open who has what responsibilities to realise climate justice in healthcare. Since ability to pay applies to healthcare organisations and to those who engage with them, first‐order and second‐order responsibilities are there to help divide up differential responsibilities. However, the agents of justice with abilities to contribute to healthcare decarbonisation is not limited to healthcare organisations and individual professionals.

### 4.1 First‐Order and Second‐Order Responsibilities

The agents of justice in healthcare are those with first‐ and second‐order responsibilities. Whilst direct interventions—like for example switching to low‐emission metered‐dose inhalers—are important, achieving net zero healthcare requires extensive, transformative structural changes and a wide variety of tasks must be performed by diverse agents. Second‐order responsibilities too, are essential for an effective transition and further expand the scope of what agents must do. Two main points can be taken from this distinction: (i) decarbonising healthcare involves more than just direct emission reductions—it also includes second‐order responsibilities; and (ii) these responsibilities are spread across a broader range of agents, not just healthcare organisations or clinicians. Next, I discuss the specific first‐order and second‐order responsibilities various agents have in reducing healthcare emissions. I do not aim to defend an exhaustive list of all first‐order and second‐order responsibilities here as decarbonising healthcare is a complicated, large‐scale process (Table 1 offers an overview).

Healthcare organisations themselves have great power to organise and coordinate the shifts in the healthcare structures, systems and processes necessary to reduce emissions. Managers, policymakers and professionals frequently have powers to effect change at a structural level. In some instances, healthcare organisations will take decisions to directly reduce emissions. For example, the anaesthetic gas desflurane has a carbon footprint many times higher than that of comparable alternatives but can be safely decommissioned as it has no distinct advantage over rivals [44]. As a wholesale and widespread change, organisations are most effective at removing desflurane from anaesthetic practice. Expecting individual anaesthetists to switch from desflurane to alternatives is likely to lead to patchy change and so organisations are best‐placed to remove desflurane [45]. A second example is shifting where healthcare is delivered to reduce GHG emissions. Organisational power will be the primary driver of providing more care in the community instead of hospitals to save emissions, not individuals. Healthcare organisations play a central role in reducing healthcare emissions but, as is clear from the tasks in Table 1, organisations cannot achieve this alone.

Action by individual clinicians is also necessary to help reduce healthcare GHG emissions. Healthcare professionals will need to alter the decisions they make and the actions they take when providing patient care to contribute to healthcare mitigation. Organisations can support, encourage and facilitate this, as I discuss below, but they hold limited capabilities to alter the decisions that professionals make. Clinicians act within certain contexts and whilst it is possible to structure these contexts to increase the chance that clinicians make more sustainable decisions, changing the structures in which decisions take place is different from changing those decisions themselves. A reciprocal relationship between healthcare structures and those who form those structures, including clinicians, exists and this has important ramifications for clinician involvement in healthcare decarbonisation.

One example is reducing overtreatment and overdiagnosis, which contribute to wasteful and unnecessary care [46, 47]. Although organisations can support clinicians by promoting sustainable models of care, providing workforce education, and fostering norms around sustainability, they face limitations in directly reducing overtreatment and overdiagnosis. Decisions that lead to overtreatment and overdiagnosis are made within the clinical interaction, where professional autonomy and patient safety are paramount. Structural changes can help, but they are often shaped by the very practices they aim to influence. Decisions that contribute to low‐value care can reproduce and reinforce the very systems that facilitate overtreatment and overdiagnosis. Clinicians who engage in defensive medicine and contribute to overtreatment reinforce the norms and systems that perpetuate these practices, creating a cycle that is difficult for organisations to break.

Beyond organisational and clinician action, reducing healthcare emissions also requires addressing broader factors. As I mentioned above, reducing healthcare emissions depends in part on reducing demand for healthcare. Tackling the social determinants of health is key to keeping people well in the first place. Governments and other sectors in society will need to take action to prevent poor health. Moreover, state action both nationally and internationally is required to decarbonise energy, transport and supply chains that all underpin most health system emissions. Further, other actors both within and outside the health sector will be required to decarbonise the supply chain, especially casting back to the fact that scope three emissions form the majority of healthcare emissions. Healthcare organisations are situated within a wider nexus of organisations some of whom will also have to take action to decarbonise healthcare.

The scope of agents with first‐order responsibilities can be expanded even further to patients. Like clinicians, patients will sometimes have to make different decisions when it comes to their healthcare. Metered‐dose inhalers provide one pertinent example where, to help healthcare reduce the emissions from inhalers, patients will have change what inhalers they rely on and how they use them [48]. Furthermore, the appropriate disposal of inhalers that contain greenhouse gases will also be important. Healthcare organisations and professionals can help support, educate, incentivise and enable patients, but reducing inhaler emissions fundamentally requires action from patients. Furthermore, wider action in society can help tackle the drivers of poor respiratory health like air pollution, and whilst this will help reduce their reliance on inhalers, it is unlikely to fundamentally alter some people's requirement for an inhaler.

I turn now to briefly consider the various ways that second‐order responsibilities might be satisfied. I have pointed to healthcare organisations, governments, individual clinicians and patients as well as others outside of healthcare as agents of justice when it comes to first‐order responsibilities. Ensuring compliance for each of these agents may mean that others take up second‐order responsibilities. I have already touched on some second‐order responsibilities, especially amongst organisations whereby creating and sustaining structures and contexts that enable individuals to reduce emissions through their practice. For example, incentives could be offered by organisations so that clinicians and patients make greener choices. Healthcare organisations are also important in creating norms that help tackle overtreatment and overdiagnosis. When it comes to supply chains, hospitals and healthcare organisations should use their relationships with suppliers as well as purchasing power to help decarbonise the supply chain and enforce environmental standards. Indeed, healthcare organisations can even have self‐directed second‐order responsibilities. For instance, a hospital could routinely record and publish data on scope one and two emissions allowing them to be tracked over time such that progress and performance can be easily assessed [25]. Professionals, patients and governments too have second‐order responsibilities. Healthcare professionals, for instance may need to lobby their institutions and governments, or engage in acts of civil disobedience, to ensure that healthcare emissions are being reduced. In addition, incentives, financial mechanisms and government targets are necessary to keep healthcare institutions on track to meet net zero. Where action on the social determinants of health is lacking, institutions, professionals and the public pressure governments to address these too.

## 5 Conclusion

In this paper, I have sought to clarify who the agents of justice are when it comes to decarbonising healthcare systems. By recognising the diverse tasks necessary to achieve climate justice in healthcare, I argued that responsibilities should be allocated based on effectiveness. This approach reveals the complexity of reducing healthcare emissions and underscores that the range of agents and the scope of their responsibilities are broader and more varied than often acknowledged. By distinguishing between first‐order and second‐order responsibilities, we gain a clearer understanding of the distribution of obligations. This also highlights that focusing solely on the direct mitigation responsibilities of healthcare professionals or organisations is insufficient for achieving meaningful progress.

## Conflicts of Interest

The author declares no conflicts of interest.

## Endnotes

1 At the time of writing, July 2025, 84 healthcare systems have made a commitment to be sustainable and low carbon.

2 It is worth noting early that the terminology the ‘agents of justice’ arises from Onora O'Neill's discussion of global justice. O'Neill was concerned with who has an obligation to fulfil the demands of global justice, especially if we cannot be confident that institutions will fulfil these demands.

3 I say ‘part of’ for several reasons. First is that mitigation does not exhaust responses to climate change and adaptation will also be necessary for healthcare systems to cope with climate change that is inevitable. Second is that net zero is necessary in emissions reductions but it is controversial whether it is also sufficient. Net zero also leaves open issues of historic responsibility as well as responsibilities to help others respond to climate change or, from a healthcare perspective, minimise the health effects of climate change. Furthermore, net zero alone does not fully describe how to reconcile providing the benefits of healthcare without reliance on emissions. Hence net zero does not tell the whole, nor a completely uncontroversial, story about achieving climate justice in healthcare. Nevertheless, it is widely thought to be a significant aspect of that story.

4 An anonymous peer reviewer raises the interesting issue of how healthcare systems might weigh emissions mitigation against other core issues of social justice within healthcare. The peer reviewer asks, “is it conceivable that in the process of decarbonizing health systems we actually make health care more expensive or less accessible?” Addressing this question adequately lies outside the scope of the paper but for the sake of clarity I will assume that net zero is sufficiently flexible in how emissions are netted out that no broader issues of distributive justice like the ones mentioned by the peer reviewer arise.

5 For comparison, breakdowns of global emissions by sector estimate that travel accounts for 16.2% of emissions, energy use in buildings is 17.5% and agriculture and land use is 18.4%.

6 I do not address whether beneficiary pays could be used to regulate healthcare decarbonisation and identify the agents of achieving climate justice in healthcare. Presumably the main beneficiaries of healthcare are patients. Neither professionals nor institutions are identified as duty‐bearers by the beneficiary pays principle and so, as these are the main agents under consideration in debates, I leave aside the beneficiary pays principle. It is however worthy of further exploration as to how to conceptualise beneficiary pays in healthcare and what it would be mean for ensuring that mitigation burdens are shared fairly.

7 Indeed, when it comes to individual duties, some authors defend a ‘principle of environmental prescribing’: *It is pro tanto wrong to choose a treatment which produces an expected amount of harm greater than any other equally clinically effective alternative unless: (1) this might undermine trust; or, (2) it significantly worsens a patient's health.* [15, 18]. This principle may also be understood in terms of ability to pay where there is a primary focus on effectively reducing healthcare emissions but limits are placed on what burdens individuals should be expected to shoulder in order to achieve sufficient healthcare decarbonisation. A continuity between moral principles regulating climate change in the doctor patient relationship and principles of justice that govern healthcare decarbonisation, may be used to question how much daylight there is between institutional or individual levels of action. I merely note this here rather than pursue this thought further.
